# The Role of Neurons in Human Health and Disease

**DOI:** 10.3390/ijms24087107

**Published:** 2023-04-12

**Authors:** Yasemin M. Akay

**Affiliations:** Department of Biomedical Engineering, University of Houston, Houston, TX 77204-5060, USA; akay481@gmail.com

Neurons are the functional units of the nervous system. They are responsible for carrying stimuli throughout the human body and help coordinate all of the necessary functions of life by using electrical and chemical signals.

The nervous system regulates immunity and inflammation. Neuroinflammation is a key component of neurological disorders and is an important therapeutic target. Neurological disorders are the conditions which affect the neurons in the human body and the rest of the nervous system, resulting in a range of symptoms. 

The specific causes of neurological diseases are different, as some neurological conditions are congenital, and the others may originate because of structural defects, degeneration, infections, tumors, or trauma. 

At any rate, all neurological diseases result from the disturbance, impairment, or deterioration of the nervous system. Symptoms of illnesses manifest themselves depending on the location, type, extent, and the dimensions of the damage.

The intention of this Special Issue is to highlight the role of neurons in human health and disease. 

In the first paper in this issue, Ugrumov et al. [1] evaluated the development of the periventricular (PeVN) in rats in the perinatal period, which is a critical period for brain formation. Their results have indicated that, in the PeVN, all types of neurons and their processes are in close relationships, and this proposes their interactive regulation by L-DOPA and DA.

According to their observation, neuron fibers deliver L-DOPA and DA to the cerebrospinal fluid, participating in the neuroendocrine regulation of the brain. This finding is important because of the L-DOPA’s effect in Parkinson’s Disease.

Liu et al. [2] studied the effects of the consumption of a high-fat diet in adolescence. The reason they have chosen this stage of development in a human’s life is that massive neural circuit wiring during adolescence causes the brain to become sensitive to the environmental effects in this period. According to the authors, this is the first study evaluating the deteriorating effects of HFD consumption in adolescence on emotion and neuroplasticity, which may be due to the excessive microglial consumption of newborn neurons. This study will help us to understand the underlying mechanism of HFD-related affective disorders in young people.

The gut–brain axis has become the main focus for many researchers lately due to its connection to both ENS and CNS, and therefore their implications in human health. In the CNS and PNS, the vascular endothelial growth factor (VEGF) mediates neuroprotective and neuroregenerative effects. Since the ENS is considered the origin of neurodegenerative diseases, it may be crucial to study the potential positive effects of the VEGF on enteric neurons. Based on the promising results of their research, Theiss et al. [3] have concluded that VEGF may have neuroprotective effects in the ENS.

The medulla oblongata is an important relay center for critical sensory, proprioceptive, and motor information. It consists of a number of nuclei all involved in different forms of essential and vital activities. Mesman et al. [4] studied the medulla oblongata to identify the progenitor domain of the origin of medullary nuclei to further explore the medulla-related symptoms of neurodevelopmental disorders. Based on the genetic defects seen in these syndromes, they suggest that they can determine which medullary nuclei might be affected and therefore targeted for the therapy of these diseases.

Functional somatic syndromes have become common in chronically ill patients presenting with a series of symptoms not pertaining to physical diseases and without a definite etiology. Stress and certain childhood traumas are now recognized as important risk factors for chronic pain conditions. Functional somatic syndromes are also highly related co-morbidities of a number of psychological and psychiatric conditions. In this review, Garvey et al. [5] aim to provide an understanding of the neuroimmune aspects of the chronic pain disorders related to the musculoskeletal and nervous systems.

In their review paper, Klimov et al. [6] point to a significant connection between the tridirectional neuroimmune network, which plays a crucial role in the communication pathways and the regulatory networks at all the systemic and local levels in both healthy and allergic rhinitis conditions. This paper focuses on neuroimmune control of the nasal mucociliary immunologically active epithelial barrier. According to Novikov, the research of pathology and treatment for atopic allergic conditions, including currently detected local structures, from the perspective of the tridirectional interaction of the neuroimmune network and discrete neuronal-immune cell units, is definitely a state-of-the-art research topic.

In this paper, Huang et al. [7] discuss the role of the immune system in the etiology of epilepsy. The involvement of ion channels in epileptogenesis and epilepsy and the complexity of their interaction with the immune system is vigorously evaluated. Moreover, there has been considerable advancement in our perception of the functional changes related to the epilepsy associated with autoimmune encephalitis. The early detection of immune-mediated epilepsy is crucial and an early intervention with immunotherapy is promising.

Astrocytes are the major homeostatic cells in the central nervous system. Their reactivation or cellular senility may have serious impacts on the immediate microenvironment, resulting in pathological results. In this review, Lazic et al. [8] outline the most recent findings related to the astrocyte reactivation and senescence, and particularly point out neurodegenerative diseases, where phenotypic differences and/or similarities of astrocytes are substantially related. They also review the recent leads related to the novel methods regarding astrocytes as possible therapeutic targets.

Alzheimer’s Disease (AD) is a multifaceted neurodegenerative condition defined by progressive cognitive deterioration, indifference, lethargy, and neuropsychiatric disorders. Neurofibrillary tangles and Aβ plaques are the major pathological indications for this disease.

In this review, Maccioni et al. [9] focus on the specific molecular pathways leading the tau protein to its pathological self-assembly, which is the main cause of Alzheimer’s Disease. 
